# Streamlined histone-based fluorescence lifetime imaging microscopy (FLIM) for studying chromatin organisation

**DOI:** 10.1242/bio.031476

**Published:** 2018-03-13

**Authors:** Alice Sherrard, Paul Bishop, Melanie Panagi, Maria Beatriz Villagomez, Dominic Alibhai, Abderrahmane Kaidi

**Affiliations:** 1 Nuclear Dynamics Laboratory, School of Cellular and Molecular Medicine, Biomedical Sciences Building, University of Bristol, Bristol, BS8 1TD, UK; 2 Wolfson Bioimaging Facility, Biomedical Sciences Building, University of Bristol, Bristol, BS8 1TD, UK

**Keywords:** ATM, DNA damage, FLIM, Chromatin, Genome, Histones

## Abstract

Changes in chromatin structure are key determinants of genomic responses. Thus, methods that enable such measurements are instrumental for investigating genome regulation and function. Here, we report further developments and validation of a streamlined method of histone-based fluorescence lifetime imaging microscopy (FLIM) that robustly detects chromatin compaction states in fixed and live cells, in 2D and 3D. We present a quality-controlled and detailed method that is simpler and faster than previous methods, and uses FLIMfit open-source software. We demonstrate the versatility of this chromatin FLIM through its combination with immunofluorescence and implementation in immortalised and primary cells. We applied this method to investigate the regulation of chromatin organisation after genotoxic stress and provide new insights into the role of ATM in controlling chromatin structure independently of DNA damage. Collectively, we present an adaptable chromatin FLIM method for examining chromatin structure and establish its utility in mammalian cells.

## Abstract (precis)

**Summary:** A streamlined and adaptable cell imaging method for quantitative analysis of chromatin organisation in mammalian cells, and its application, reveal a new role for ATM in regulating chromatin structure.

## INTRODUCTION

In the nucleus, DNA is packaged into chromatin structures (Richmond and Davey, 2003) that determine the activity of genomic DNA in space and time (Bickmore, 2013; Dekker et al., 2017), and may also contribute to non-genetic functions of the genome (Bustin and Misteli, 2016). Such chromatin organisation is underpinned by regulatory epigenetic mechanisms, including histone modifications (Kouzarides, 2007). Visually, interphase chromatin appears to exist in two clearly distinct states: open euchromatin and condensed heterochromatin (Bickmore and van Steensel, 2013). Although these chromatin states seem to be stable at steady-state conditions, they undergo dynamic reorganisation during genome transduction processes such as transcription (Therizols et al., 2014; Wang et al., 2014), or DNA repair (Lukas et al., 2011; Polo and Almouzni, 2015). Therefore, experimental approaches that enable quantitative analysis of global and regional chromatin compaction states will likely advance our understanding of the principles that govern genome organisation and regulation.

Advanced cell imaging techniques have proven instrumental in the study of chromatin organisation in intact cells. Cryo-electron tomography (Ou et al., 2017) and super-resolution light microscopy methods (Boettiger et al., 2016; Ricci et al., 2015) have provided unprecedented insights into the spatial and temporal organisation of chromatin. These powerful methods point to a spectrum of chromatin compaction states with a degree of heterogeneity (Boettiger et al., 2016; Ou et al., 2017; Ricci et al., 2015). In addition, techniques such as fluorescence correlation spectroscopy (FCS) (Toh et al., 2015) and fluorescence anisotropy (Bhattacharya et al., 2009), which involve the use of fluorescently tagged histones (Mora-Bermúdez and Ellenberg, 2007), have allowed analysis of chromatin dynamics.

To determine chromatin compaction states, Forster (fluorescence) resonance energy transfer (FRET) can be used, where histones are tagged with FRET-compatible fluorescent proteins. FRET measurements can in turn be quantitatively determined by fluorescence lifetime imaging microscopy (FLIM), wherein the lifetime of the donor fluorophore (e.g. GFP) is comparatively measured in the absence or presence of an acceptor probe (e.g. mCherry) (Ishikawa-Ankerhold et al., 2012). This chromatin-FLIM-FRET approach has been previously elegantly performed and validated in live human HeLa cells (Llères et al., 2009). However, a key limitation of this previous approach is low scalability as well as difficulties in its applicability, particularly due to the method of generating mammalian cell lines with appropriate distribution of fluorescently tagged histones, and the requirement for a specialised microscope (Llères et al., 2009).

Here, we report further developments to streamline a chromatin FLIM protocol that is simpler and scalable. We provide a detailed and adaptable experimental pipeline that allows faster data acquisition, quality-controlled data analysis using open source software, and experimental reproducibility. As well as validating our chromatin FLIM, we applied this method to examine the regulation of chromatin organisation in response to DNA damage and by the DNA damage response machinery.

## RESULTS

**Fig. 1.**
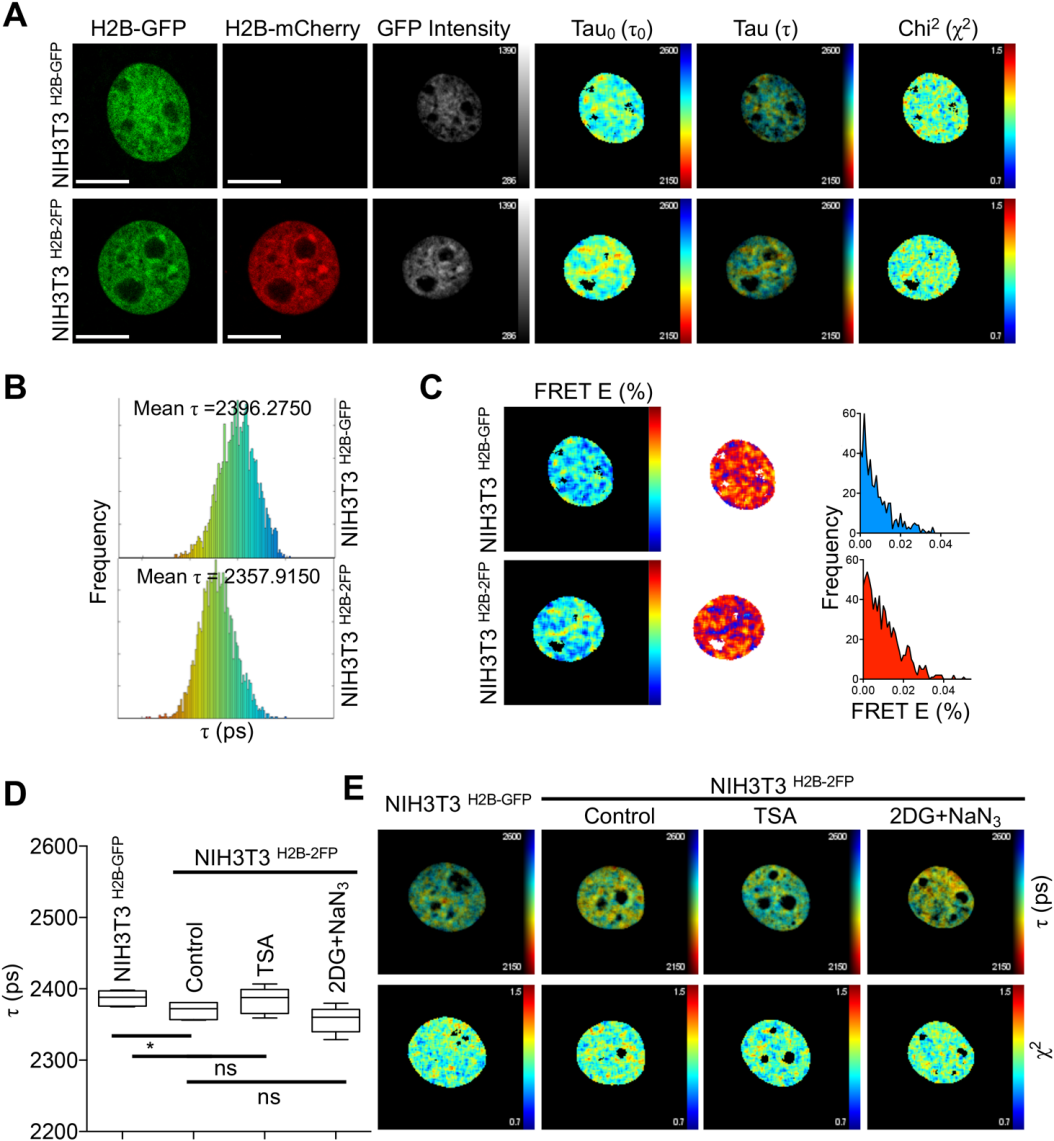
**Initial establishment of chromatin FLIM assay in NIH3T3 cells.** (A) Examples of the FLIM measurement pipeline in the indicated NIH3T3 cells. Images for H2B-GFP and H2B-mCherry were acquired and FLIM data analysis was performed on FLIMfit software that generates the corresponding GFP intensity, GFP fluorescence lifetime Tau_0_ (τ_0_), merged fluorescence lifetime Tau (τ) and chi-squared (χ^2^) maps (please note that the raw images have been re-sized for publication). (B) The distribution of Tau (τ) values on a pixel-by-pixel basis of the same nuclei shown in A. (C) Representative FRET efficiency (FRET E) maps (presented with two different colour scales) derived from the corresponding FLIM maps in A, with pixel-based FRET efficiency distributions. (D) Quantification of fluorescence lifetime (τ) in the indicated cells and growth conditions. Data are expressed in picoseconds (ps) as mean τ±s.d., *n*≥5, *P=0.0422; ns, not significant, Student's *t*-test. (E) Example fluorescence lifetime (τ), chi-squared (χ^2^) maps from D. Scale bars: 10 μm.

In pursuit of developing a simple and scalable chromatin FLIM experimental system, we used lentiviral transduction to derive NIH3T3 cell lines expressing H2B-GFP alone (NIH3T3^H2B-GFP^) or in combination with H2B-mCherry (NIH3T3^H2B-2FP^). These fluorescently tagged histones were stably expressed (Fig. S1A) and were resistant to extraction with cytoskeleton (CSK) buffer prior to cell fixation (Fig. S1B), indicative of their incorporation into chromatin (Polo et al., 2010). The expression of these histones had little effect on cell proliferation (Fig. S1C) and DNA replication (Fig. S1D), confirming that the expression of these fluorophores does not affect cell growth. Next, we conducted experiments to measure H2B-GFP fluorescence lifetime in the corresponding cells after paraformaldehyde fixation. The data were analysed using FLIMfit (Warren et al., 2013), an open-source software (see Materials and Methods for details), which generates a corresponding fluorescence lifetime (Tau_0_: τ_0_, Fig. 1A) map on a pixel-by-pixel basis. For increased accuracy, a merged map is generated wherein each pixel is represented with a colour code, dictated by its fluorescent lifetime value, and its brightness determined by the GFP intensity during FLIM data acquisition (Fig. 1A). This merged map is informative as it depicts the fluorescence lifetime (Tau: τ, Fig. 1A) and enables the visualisation of a spectrum of chromatin structures within the nucleus. FLIMfit software also provides graphical representation of chi-squared (χ^2^) values, a statistical test that indicates the extent of variation between the actual FLIM measurements and the data-fitting model, thereby providing a quality control for validating FLIM data analyses (Fig. 1A). To this end, we found that the mean H2B-GFP fluorescence lifetime was higher in NIH3T3^H2B-GFP^, compared to NIH3T3^H2B-2FP^, at the single nucleus level (Fig. 1A). This difference in fluorescence lifetime can also be observed as a shift in the distribution of H2B-GFP fluorescence lifetime values of each pixel (Fig. 1B), and is consistent with specific FRET from H2B-GFP (donor) to H2B-mCherry (acceptor) when they are co-expressed in NIH3T3^H2B-2FP^ cells (Fig. 1C). We also extended the utility of the chromatin FLIM approach to human retinal pigment epithelial-1 (RPE1) cells (Fig. S2A,B), thus demonstrating that this method is an adaptable experimental approach for performing chromatin FLIM in fixed cells as a readout for the extent of FRET between two fluorescently tagged histones.

A challenge in performing chromatin FLIM is the ability to conduct FLIM measurements in multiple mammalian cells (Llères et al., 2009). Addressing this limitation would not only provide experimental robustness through statistical power but would also allow the identification of potential heterogeneity within a population of cells. Accordingly, we analysed H2B-GFP fluorescence lifetime in multiple fixed NIH3T3^H2B-GFP^ and NIH3T3^H2B-2FP^ cells from the same experiment. As shown (Fig. 1D), H2B-GFP fluorescence lifetime in NIH3T3^H2B-GFP^ was significantly higher than in NIH3T3^H2B-2FP^, thus extending and confirming our single-cell analyses.

Changes in H2B-GFP fluorescence lifetime in NIH3T3^H2B-2FP^ cells can be used to infer the proximity between H2B-GFP and H2B-mCherry (typically in the range of ∼1-10 nm), and therefore it relates to the extent of chromatin compaction (Llères et al., 2009). When chromatin is in its de-compact state, nucleosomes are spaced apart, leading to higher fluorescence lifetime of the donor fluorophore, due to a decrease in FRET from to the donor to acceptor fluorophores (Ishikawa-Ankerhold et al., 2012). When chromatin is in its compact state, nucleosomes are closer to one another, leading to an increase in FRET and a decrease in the donor fluorescence lifetime. As such, we used growth conditions to alter chromatin compaction in interphase NIH3T3^H2B-2FP^ cells. Initially, we found that inducing chromatin relaxation using the HDAC inhibitor Trichostatin A (TSA) did not significantly affect H2B-GFP fluorescence lifetime at a cell population level (Fig. 1D). Similarly, increasing chromatin compaction using 2-deoxyglucose (2DG) and sodium azide (NaN_3_)-mediated ATP depletion had no significant effect on H2B-GFP fluorescence lifetime in a cell population (Fig. 1D). However, when examining H2B-GFP fluorescence lifetime at the single-cell level, we could detect an increase after TSA treatment and a decrease after 2DG+NaN_3_ (Fig. 1E), consistent with a previous study (Llères et al., 2009).

**Fig. 2.**
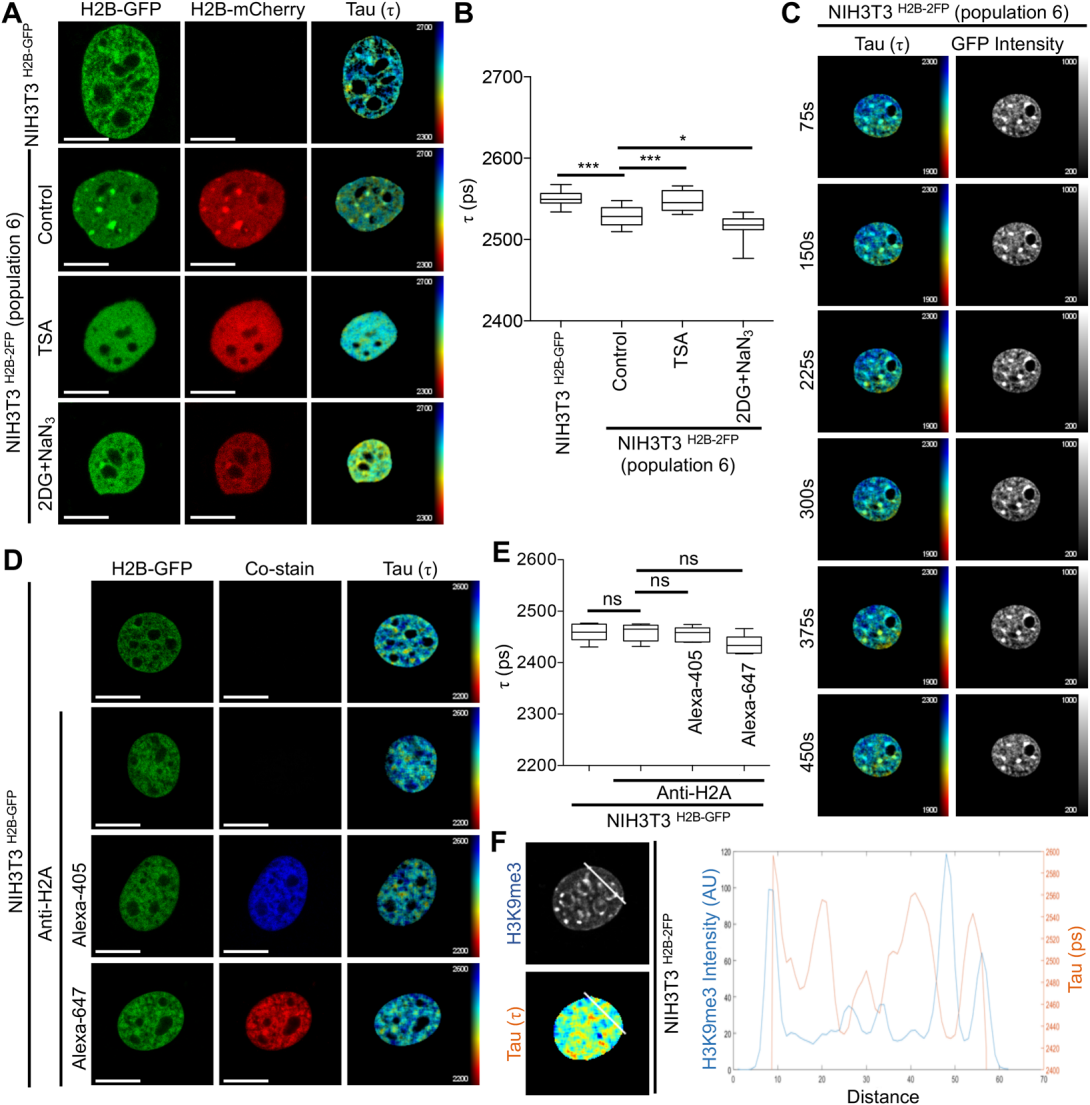
**Streamlining chromatin FLIM assays.** (A) Representative examples of FLIM measurements conducted in the indicated cells and conditions. (B) Quantifications of lifetime (τ) data from A expressed as mean τ±s.d., *n*≥15, *P=0.0272, ***P≤0.001, Student's *t*-test. (C) Live-cell chromatin FLIM in NIH3T3^H2B-2FP^ at the indicated time intervals. (D) Example of GFP-H2B lifetime (τ) in fixed NIH3T3^H2B-2FP^ co-stained with anti-H2A using AlexaFlour-405 or AlexaFluor-647. (E) Quantification of τ data from D expressed as mean τ±s.d., *n*≥17; ns, not significant, Student's *t*-test. (F) Analysis of line profiles of H3K9me3 (blue) staining and a parallel FLIM map (orange) showing that regions high in H3K9me3 correspond to lower lifetime (τ). Scale bars: 10 μm.

Given that both TSA and 2DG+NaN_3_ treatments are well documented to alter chromatin condensation state, our observed heterogeneity in H2B-GFP fluorescence lifetime between cells could be attributed to the relative levels of H2B-GFP and H2B-mCherry expression in NIH3T3^H2B-2FP^ cells. To test this, we reasoned to perform fluorescence activated cell sorting (FACS) to generate different NIH3T3^H2B-2FP^ cell subpopulations with varying expression levels of H2B-GFP and H2B-mCherry. We sorted eight different subpopulations of NIH3T3^H2B-2FP^ (Fig. S3A) and performed FLIM experiments to determine the extent of heterogeneity in H2B-GFP fluorescence lifetime. We observed that subpopulations 3 and 6 had the lowest degree of variation in H2B-GFP fluorescence lifetime (Fig. S3B). By using subpopulation 6 in FLIM experiments, we found that TSA treatment resulted in a significant increase in H2B-GFP fluorescence lifetime at a population level and that 2DG+NaN_3_ decreased H2B-GFP fluorescence lifetime (Fig. 2A,B). Therefore, by controlling the expression levels and homogeneity of H2B-GFP and H2B-mCherry, we present improvements that allow quantitative analysis of chromatin structure using FLIM in fixed cells at a population level, with comparable results to previously published data from single cells (Llères et al., 2009).

As using FACS is not suitable for all cell types, and to increase the versatility of our chromatin FLIM method, we applied an alternative approach using cultured primary neurons (not amenable to FACS) as an example. To this end, we used pre-extraction with CSK buffer (Kaidi et al., 2010) prior to cell fixation to minimise variation in the levels of H2B-GFP and H2B-mCherry proteins, which we identified earlier to be a critical factor in the feasibility of our FLIM approach. Accordingly, pre-extraction allowed us to measure fluorescence lifetime changes as well as chromatin relaxation induced by TSA, in both unsorted NIH3T3 cells (Fig. S3C,D), as well as in primary neurons (Fig. S3E,F).

Upon the development of this experimental system, we next optimised FLIM data acquisition, to increase the speed at which FLIM measurements can be taken in live cells. By adjusting acquisition parameters (see Materials and Methods), we were able to conduct chromatin FLIM in live cells at time intervals of 75 s (Fig. 2C), compared to 200 s reported previously (Llères et al., 2009). This provides a scope for more rapid chromatin rearrangements to be detected, and extends previous elegant work that allowed chromatin-based FLIM in live cells (Llères et al., 2009, 2017).

Another advantage of FLIM in fixed cells would be the possibility of combining FLIM with immunofluorescence staining. Indeed, this could be useful when investigating changes to chromatin organisation, for example within specific nuclear compartments or in response to cellular stimuli that can both be monitored by using immunocytochemistry. However, an important consideration would be to ensure that fluorescent secondary antibodies do not interfere with chromatin FLIM. Accordingly, we stained for histone H2A in FACS-sorted NIH3T3^H2B-2FP^ cells using either AlexaFluor-405 or AlexaFluor-647 secondary antibodies, followed by measuring H2B-GFP fluorescence lifetime. The results revealed that the presence of AlexaFluor-647 interferes slightly with H2B-GFP fluorescence lifetime (Fig. 2D,E), whereas using AlexaFluor-405 had less discernible effect (Fig. 2D,E). This important control suggests that parallel AlexaFluor-405 staining is compatible with performing FLIM experiments using the GFP-mCherry protein pair. Having validated a chromatin FLIM method that can be combined with conventional immunocytochemistry, we tested this utility of this approach is quantifying fluorescence lifetime is specific chromatin environments. To this end, and given that H3K9me3 is enriched in the highly compacted pericentromeric heterochromatin, by plotting line profiles we could show that H3K9me3 levels inversely correlate with H2B-GFP fluorescence lifetime, as expected. This further validated our chromatin FLIM for detecting specific chromatin environments through combination with immunocytochemistry (Fig. 2F).

**Fig. 3.**
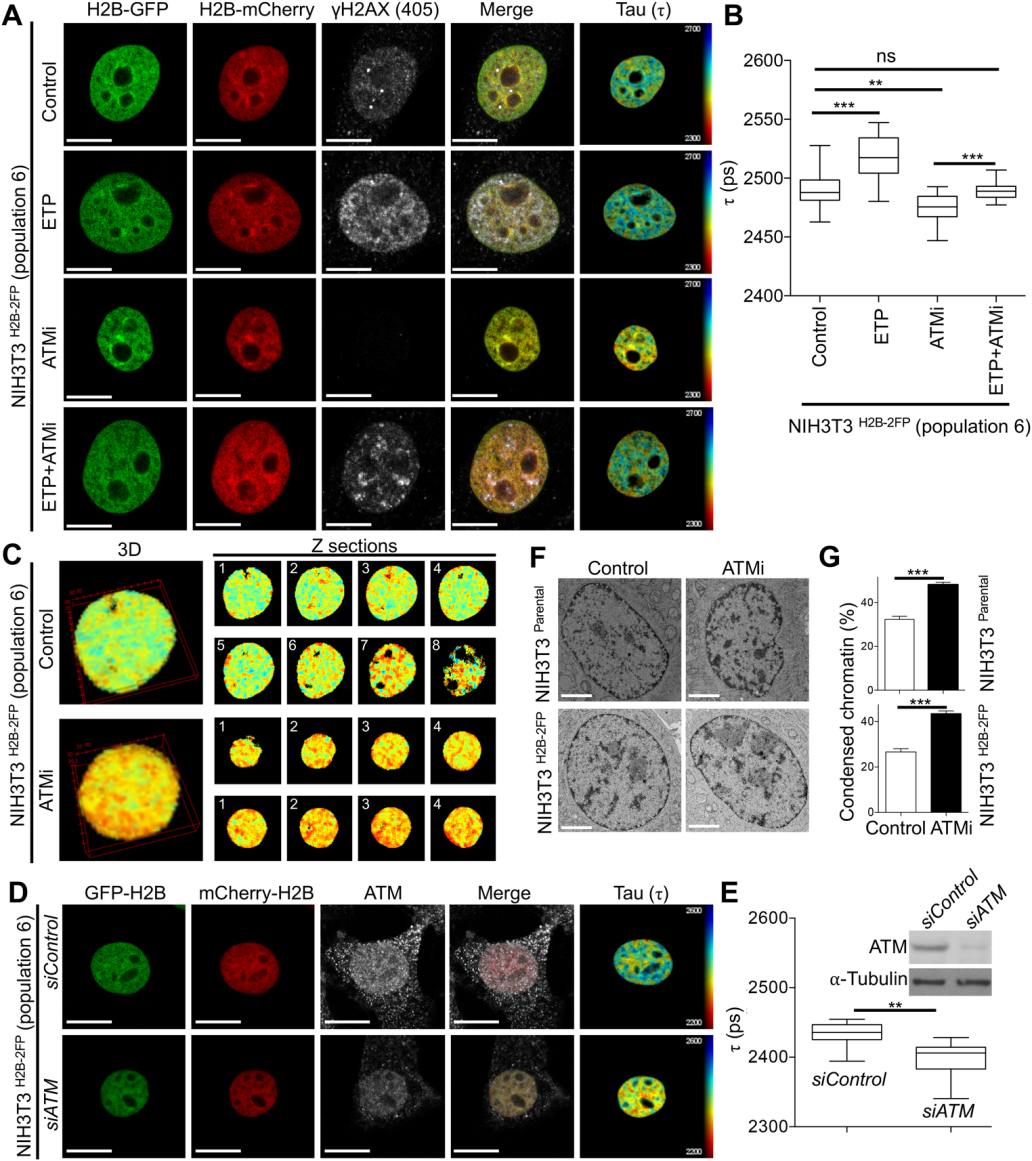
**ATM regulates chromatin compaction states.** (A) Representative examples of FLIM measurements conducted in the indicated cells that were treated as indicated and co-stained for γH2AX. (B) Quantifications of τ data from A expressed as mean τ±s.d., *n*≥18, **P=0.0014, ***P≤0.001; ns, not significant, Student's *t*-test. (C) Example of 3D-FLIM in NIH3T3^H2B-2FP^ cells treated with ATMi; the right panels are different Z sections from C (with lower zoom). (D) Representative examples of FLIM measurements conducted in indicated cells after siRNA-mediated depletion of ATM and co-stained for ATM (to confirm knockdown). (E) Corresponding quantifications of fluorescence lifetime from D, *n*≥10, **P=0.0024, Student's *t*-test. The immunoblot confirms ATM knockdown in a parallel experiment. (F) Electron microscopy images of cryopreserved cells after treatment of ATM inhibition. (G) Corresponding quantifications of condensed chromatin from F, as described in the Materials and Methods, *n*≥20, ***P≤0.001, Student's *t*-test. Scale bars: 10 μm in A and D, 2 μm in F.

With these new methodological advances, we applied this approach to visualise chromatin structure in response to DNA damage, while at the same time identifying DNA damage markers. Accordingly, we treated NIH3T3^H2B-2FP^ cells with the topoisomerase II inhibitor etoposide (ETP) and confirmed DNA damage induction by the detection of H2AX-phosphorylation at S139 (γH2AX) and KAP1-phospho-S824 (pKAP1) (Fig. 3A; Fig. S4A). When analysing DNA-damaged γH2AX positive cells, we found that DNA damage resulted in increased H2B-GFP fluorescence lifetime (Fig. 3A,B), suggestive of increased chromatin relaxation after DNA damage (Takahashi and Kaneko, 1985). Inhibition of the DNA damage kinase ATM (Fig. S4B) markedly reduced etoposide-induced chromatin de-compaction (Fig. 3A,B), consistent with previous reports of ATM-dependent chromatin relaxation after DNA damage (Caron et al., 2015; Ziv et al., 2006). These results highlight the usefulness of our chromatin FLIM method for detecting and validating known structural changes of chromatin in response to genotoxic stress.

Notably, we observed that ATM inhibition alone resulted in a marked decrease in H2B-GFP fluorescence lifetime (indicative of increased chromatin compaction) both in 2D (Fig. 3A,B) in 3D (Fig. 3C). Similarly, we found that siRNA-mediated depletion of ATM significantly reduced H2B-GFP fluorescence lifetime, pointing to a specific role for ATM in regulating chromatin structure (Fig. 3D,E). To further confirm this effect of ATM inhibition increasing chromatin compaction, we used electron microscopy to quantify chromatin density in cryo-preserved cells, as we described previously (Baarlink et al., 2017). To this end, we found that ATM inhibition quantitatively increases chromatin density both in parental NIH3T3 (NIH3T3^parental^) and NIH3T3^H2B-2FP^ cells (Fig. 3F,G). These electron microscopy results not only corroborate our FLIM data (thus method) but also confirm that expression of fluorescently tagged histones does not interfere with chromatin structural responsiveness in NIH3T3^H2B-2FP^. Of note, we observed that inhibition of CHK2 – a downstream target of ATM in the DNA damage response (DDR) (Matsuoka et al., 2000) – did not increase chromatin compaction (Fig. S4B), suggesting that ATM's ability to regulate chromatin structure may be – at least in part – distinct from its canonical role in DDR signalling.

**Fig. 4.**
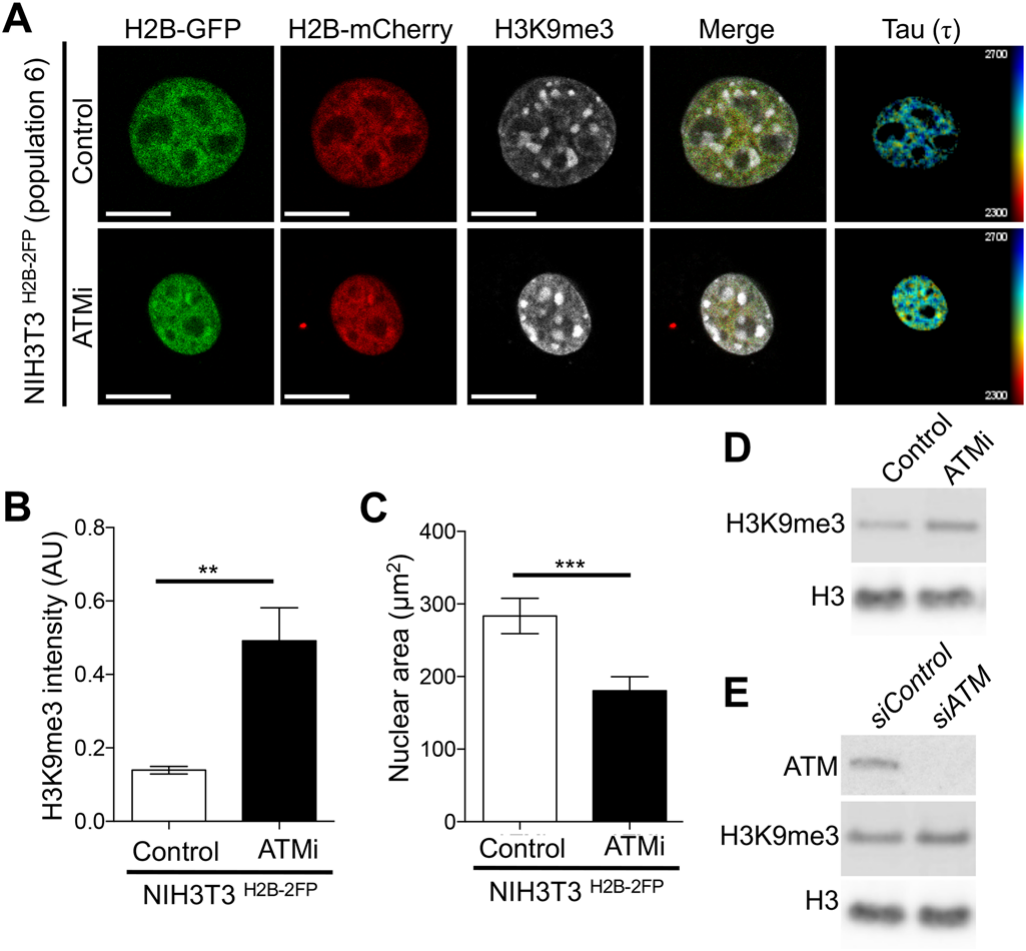
**ATM regulates chromatin compaction and H3K9me3.** (A) Representative examples of FLIM measurements conducted in the indicated cells after treatment with ATMi and co-staining for H3K9me3. (B,C) Quantifications of H3K9me3 fluorescence intensity (B) and nuclear area (C) from A, expressed as mean τ±s.d., *n*=20, **P=0.0044, ***P=0.0007, Student's *t*-test. (D,E) Representative immunoblot analysis of H3K9me3 after ATM inhibition (D) or ATM depletion (E), showing increased levels of H3K9me3, using total histone H3 as a loading control. Scale bars: 10 μm.

The increased chromatin compaction after ATM inhibition could be attributed to loss of histone lysine acetylation. However, treatment with the lysine deacetylase inhibitor TSA, which by itself increased chromatin relaxation (Fig. S5), did not fully alleviate the higher chromatin compaction in ATM inhibited cells (Fig. S5). These observations prompted us to hypothesise that basal ATM activity may somewhat be required for maintaining chromatin organisation in interphase cells, in a manner that mechanistically involves the regulation of condensed heterochromatin. To further investigate this and gain some mechanistic insights, we performed FLIM measurements and analysed the heterochromatic histone mark H3K9me3 (histone H3 lysine 9 trimethylation) (Bannister et al., 2001) in the same cells, while also measuring nuclear area. These concurrent analyses revealed that ATM inhibition resulted in a marked increase in H3K9me3 fluorescence intensity (Fig. 4A,B), and a significant decrease in nuclear area (Fig. 4C), consistent with reduced fluorescence lifetime (Fig. 4A) and increased chromatin compaction. We could also detect an increase in H3K9me3 by immunoblotting after ATM inhibition (Fig. 4D) or depletion by siRNA (Fig. 4E). Collectively, these findings reveal a – hitherto unappreciated – role for ATM basal activity in regulating chromatin structure, in a manner involving the heterochromatic mark H3K9me3.

## DISCUSSION

Here, we report further developments that enable streamlining of chromatin FLIM for visualising chromatin structure and compaction states in live and fixed mammalian cells. We present our method in a gradual way, wherein we highlight troubleshooting steps, validations, and quality controls for the purpose of widening utility and application of chromatin FLIM. In so doing, our chromatin FLIM method extends on previous elegant work (Llères et al., 2009, 2017), specifically through: (i) requiring simpler microscopy setup (confocal with pulse-laser, instead of a multiphoton microscope); (ii) increasing the speed of FLIM data acquisition; (iii) using an open-source software (FLIMFit) for data fitting and analysis; (iv) being scalable to cell populations; (v) adaptability to different cell types; and (vi) combination with immunofluorescence. Additionally, our method is amenable to automation for the purpose of screening, which may be useful to identify agents and/or factors that influence chromatin structure, with potential to reveal the mechanisms underlying genome organisation in space and/or time. This is particularly relevant given that FLIMfit software is part of the Open Microscopy Environment (OME) (Jason, 2013) that seamlessly integrates large data sets from phenotypic screens. Relevant to this, through the use of pixel-based segmentation in combination with antibody labelling, our chromatin FLIM method detected higher chromatin compaction within H3K9me3-rich chromatin, suggesting that our chromatin FLIM can reveal specific chromatin environments based on their compaction status. This could in turn be adapted to provide insights into the association of condensed/de-condensed chromatin with subnuclear domains, or chromosome territories, thus increasing our understanding of genome compartmentalisation (Bickmore and van Steensel, 2013).

Chromatin FLIM data can be used to calculate FRET efficiency (Padilla-Parra et al., 2015) (Zeug et al., 2012) between fluorescently tagged histones, thereby providing another readout for chromatin compaction states (Llères et al., 2009). Indeed, we were able to conduct such conversion, and show that nuclear regions of lower H2B-GFP fluorescence lifetime are associated with higher FRET efficiency (due to proximity between H2B-GFP and H2B-mCherry), as expected. However, we reasoned to present our data as pixel-based fluorescence lifetime maps (for single cells), and distributions of mean pixel-fluorescence lifetimes from different nuclei (as box plots). One of the strengths of lifetime measurements is their independence from the intensity of the signal and the inherent ratiometric nature of the data fitting process (Padilla-Parra et al., 2015). Also, lifetime measurements for a fluorophore can be compared across different microscopes. Calculation of FRET efficiency can be useful in identifying specific chromatin environments (e.g. highly condensed heterochromatin); however, this invariably relies on a biased thresholding of FRET efficiency (Llères et al., 2009).

Beyond the streamlining of chromatin FLIM, we show its utility in detecting chromatin de-compaction after DNA damage in intact cells, wherein we show genome-wide chromatin relaxation (and increased fluorescence lifetime) after etoposide treatment, and found that this effect is partly dependent on ATM activity. These FLIM findings confirm previous studies that used biochemical Micrococcal nuclease (MNase) accessibility assays (Ziv et al., 2006), chromatin-immunoprecipitation (ChIP) (Berkovich et al., 2007), or electron spectroscopic imaging (ESI) (Kruhlak et al., 2006), when examining chromatin de-condensation after ionising radiation and/or site-specific DNA double-strand breaks. While the ability of ATM to regulate global chromatin de-condensation after DNA damage has been linked to KAP1 phosphorylation (Ziv et al., 2006), local chromatin de-compaction at DNA double-strand breaks involves the cooperative function of ATM with NBS1 (Berkovich et al., 2007). ATM-dependent chromatin de-compaction mechanisms are particularly pertinent given the role of ATM in promoting DNA repair within condensed heterochromatin (Goodarzi et al., 2008).

Of particular interest, we reveal that ATM inhibition results in a genome-wide increase in chromatin compaction in a manner that is mechanistically associated with higher levels of H3K9me3. This suggests a role for ATM (presumably its basal activity) in chromatin surveillance, and likely independently of DNA damage. Indeed, this is in line with previous studies highlighting the crosstalk between chromatin structure and DNA damage signalling independently of DNA breaks per se (Bakkenist and Kastan, 2003; Burgess and Misteli, 2015; Burgess et al., 2014; Kaidi and Jackson, 2013). Accordingly, it is possible that the reported ATM-dependent transcriptional inhibition (Kruhlak et al., 2007; Shanbhag et al., 2010) may somewhat be mediated through ATM regulation of chromatin compaction. Therefore, the prospect of future investigations into ATM as a regulator and/or surveyor of chromatin organisation, beyond its role in the DNA damage response, will likely provide new insights into the relationship between genome structure and stability. Such ATM function may help to explain its role in neurodegeneration (Jackson and Bartek, 2009), wherein heterochromatin appears to be dysregulated (Frost et al., 2014). Also, given its homology with ATR (Awasthi et al., 2016), which has been reported to signal during cellular mechanotransduction (Kumar et al., 2014), it is possible that ATM may regulate chromatin compaction states during mechanical cell signalling events that are known to modulate genome structure and function (Miroshnikova et al., 2017; Shivashankar, 2011), and perhaps contribute to the structural non-genetic function of the genome (Bustin and Misteli, 2016).

In summary, we have furthered the development of a validated and versatile method to assess chromatin compaction states in intact cells, and applied this method to provide new insights into the regulation of chromatin compaction by ATM. Given the importance of chromatin structure in the control of genome transduction processes such as transcription and DNA repair, we envisage that future research may take advantage of a chromatin FLIM approach – as described here – to further advance mechanistic understanding of spatiotemporal control of genome organisation and function, both in health and disease.

## MATERIALS AND METHODS

### Cell culture, treatments, and transfection

NIH3T3 cells were cultured in in Dulbecco's Modified Eagle's Medium (DMEM) and RPE1 cells in DMEM-F12 Ham (Gibco), containing 2 mM glutamine (Sigma-Aldrich), 100 units/ml Penicillin (Gibco), 100 g/ml Streptomysin (Gibco) and 10% foetal bovine serum (FBS) (Gibco). Cells were maintained at 37°C in 5% CO_2_ dry incubators. For FLIM experiments, cells were seeded in glass µ-dishes (ibidi, Thistle Scientific, Glasgow, UK). Cells were treated with 1 µM TSA (Sigma-Aldrich) for 16 h, 50 mM 2-deoxyglucose (Sigma-Aldrich) and 10 mM sodium azide (Sigma-Aldrich) for 30 min, and 10 µM ATM inhibitor ATMi (Abcam, KU-55933) or 1.5 µM CHK2i (Sigma-Aldrich, C3742) for 18 h before treatment with 10 µM etoposide (Sigma-Aldrich) for 1 h. All cells are mycoplasma-frees routinely tested for mycoplasma contamination using PCR.

Primary hippocampal neuronal cultures were prepared from embryonic day (E) 18 rats and maintained using standard conditions. Neurons were grown on 35 mm glass-bottom dishes (ibidi). Day-*in-vitro* (DIV) 2 neurons were infected with H2B-GFP and H2B-mCherry lentiviruses. On DIV6, neurons were treated with 55 mM KCl for 3 min to increase histone exchange. On DIV7, neurons were then pre-extracted and fixed as described. Animal care and all experimental procedures were conducted in accordance with UK Home Office and University of Bristol guidelines.

Transfection with siRNA was conducted using Lipofectamine RNAi Max, flowing the manufacturer’s protocol. For ATM depletion we used the following: sense: CAUCUAAUGGUCUAACGUA[dTdT]; antisense: UACGUUAGACCAUUAGAUG[dTdT].

### Antibodies

Antibodies were obtained from Cell Signaling Technology (H2A, 12349P; H3K27me3, 9733S; DyLight-680, 5470 and Dylight-800, 5151), Abcam (AlexaFluor-405, ab175651; H3K9me3, ab8898; H2A.X, ab124781; KAP1, ab10483; KAP1-pS824, ab70369; HP1γ, ab10480; H3K9me2, ab1220; and ATM, ab32420), Invitrogen (AlexaFluor-647, A21245), Millipore (γH2A.X, 05-636; HP1α, 05-689), Roche (GFP, Roche-11814460001) and MBL Life Science (Caltag Medsystems, Buckingham, UK) (mRFP, PM005)

### Immunofluorescence staining

Cells were fixed in 4% paraformaldehyde for 10 min, permeabilised in 0.1% Triton X-100 for 10 min, and blocked in 1% BSA in PBS for 30 min. For pre-extraction experiments, cells were incubated in CSK buffer (10 mM PIPES, pH 6.8, 100 mM NaCl, 300 mM sucrose, 3 mM MgCl_2_, 1 mM EGTA, 0.5% Triton X-100) for 5 min before fixation. Cells were incubated with primary antibody for 2 h, and secondary antibody for 45 min, both at room temperature.

### Production of lentivirus

To produce lentivirus, the *PGK-H2B-GFP* and *PGK-H2B-mCherry* vectors were obtained from Addgene (Cambridge, MA, USA). Each of these plasmids was co-transfected with helper plasmids into HEK293T packaging cells. Lentivirus particles were recovered using standard protocols.

### FACS

H2B-GFP- and H2B-mCherry-expressing NIH3T3 cells were isolated using FACS, and sorted into eight populations (see FACS plots). Viable cells were identified based on light scatter and the exclusion of propidium iodide (PI). In addition, single-cell gating was used to exclude doublets and aggregated cells. H2B-GFP- and H2B-mCherry-expressing cells were sorted using 488 nm laser excitation and 510-550 nm emission and 552 nm excitation with 600-620 nm emission, respectively, using a Becton Dickinson InFlux cell sorter (BD Biosciences) running BD Software version 1.2.

### Flow cytometry

Cells were treated with 10 μm EDU for 30 min, before being fixed and stained following the EDU click chemistry protocol (C10635). Cells were stained with PI (40 μg ml^−1^), and analysed on a flow cytometer (Novocyte, San Diego, CA, USA). Data were prepared using FlowJo (https://www.flowjo.com/).

### FLIM data acquisition

FLIM was performed on cells growing on glass µ-dishes (ibidi). In the case of fixed samples, no mounting medium was used, and FLIM was performed in PBS. Fluorescence lifetime images were acquired on a Leica TCS SP8 system attached to a Leica DMi8 inverted microscope (Leica Microsystems, Wetzlar, Germany). Excitation was provided by a white light laser with a repetition rate of 20 MHz and an acousto-optical beam splitter (AOBS) selected an excitation wavelength of 488 nm. Excitation continued for 75 s/FLIM measurement/focal plane. Images were acquired using a 63×1.4 NA oil immersion objective. Fluorescence of the H2B-GFP was detected using a hybrid detector operating in photon counting mode over an emission range of 495–530 nm. A notch filter centred on 488 nm minimised any laser scatter into the detector. Time-resolved data were acquired through use of a PicoHarp 300 TCSPC module (PicoQuant, Berlin, Germany) controlled through SymPhoTime64 software (PicoQuant). FLIM Images were acquired with 512×512 pixels and 4096 time bins. For live-cell experiments, the system was maintained at 37°C/5% CO_2_. In 3D experiments, FLIM measurements were taken at a minimum of eight focal planes, 1 µm apart.

### FLIM data fitting

Fitting of FLIM images was performed with the FLIMfit software tool (version 5.0.3) developed at Imperial College London. Temporal binning of the fluorescence decays was performed prior to fitting, resulting in 401 time bins per decay and the images were spatially binned 4×4 to ensure sufficient photons were present per pixel prior to the fitting of the data. Fitting of the fluorescence images was then performed pixelwise with a single exponential model on all pixels above an intensity threshold of 200 photons with a 5×5 smoothing kernel applied, allowing spatial variations in fluorescence lifetime to be visualised. The instrument response function (IRF) was measured by imaging a solution of 1 µM rhodamine 6G with the same settings as data acquisition and then using the ‘Estimate IRF’ function within the FLIMfit software to extract the IRF.

### Calculation of FRET efficiency

Mean FRET efficiency was calculated from pixel-based fluorescence lifetime measurements (τ) wherein the FRET efficiency (E), E=1−(τ_pixel_/τ_mean_).

### Generation of parallel line profiles from FLIM maps and immunofluorescence images

As the images of the H3K9me3 channel and the GFP fluorescence lifetime channel did not overlap (due to scaling from FLIMfit), image registration was used to correct for any misalignment. H3K9me3 channel image was scaled to be 128×128 pixels using bicubic interpolation in ImageJ (https://imagej.nih.gov/ij/) and rotated to align with the lifetime image. These rotated and scaled images were loaded into MATLAB (MathWorks) and registered with an affine transform to ensure best alignment. Data were saved from MATLAB and then a line profile of 2 pixels width was drawn in ImageJ through centromeres, identified as focal regions showing high intensity in the H3K9me3 channel. The same line was then copied onto the GFP lifetime map and a corresponding line profile was extracted.

### Imaging data analysis

Fiji software (Schindelin et al., 2012) was used to segment the nucleus, based on the H2B-GFP channel. The size of the segmented nuclear area (in pixels), and the intensity of H3K9me3 within it were then quantified using Fiji software.

### Electron microscopy

Cells were placed into a 0.1 mm gold membrane carrier and high pressure frozen. Samples were then freeze-substituted in a freeze-substitution acetone mix, containing 0.1% uranyl acetate and 1% osmium tetroxide. During this procedure, samples were first held at −90°C, then brought to 0°C, over a period of 18 h. Then, 70 nm sections were cut using an ultratome, which were stained with uranyl acetate and lead citrate, and images were taken at 2900× magnification on a FEI Tecnai 12 transmission electron microscope (FEI, Eindhoven, Netherlands), operated at 120 kV.

Quantification of condensed chromatin was performed as described previously. Briefly, nuclei and nucleoli were segmented to generate a binary mask of the nucleoplasm. Condensed chromatin was then semi-automatically segmented across the nucleoplasmic region using the WEKA Trainable Segmentation plugin for ImageJ/Fiji 40. Classification was based on the Gaussian blur, Sobel filter, Hessian, Difference of Gaussians and membrane projections metrics using the built-in fast random forest algorithm. Condensed chromatin distribution was subsequently analysed in the segmented images using a custom ImageJ/Fiji macro, which measured the total condensed chromatin area and perimeter, as well as the area fraction of condensed chromatin, as a proportion of the total nucleoplasmic area.

### Statistics

Statistical analysis was performed with GraphPad Prism, using Student's *t-*test to compare selected pairs, as indicated.

## Supplementary Material

Supplementary information
