# Supplementary material for: Streamlined histone-based fluorescence lifetime imaging microscopy (FLIM) for studying chromatin organisation
Source: Biol Open. 2018 Mar 13;7(3):bio031476. doi: 10.1242/bio.031476 (PMC5898265; doi:10.1242/bio.031476)
Supplement: Supplementary information [file biolopen-7-031476-s1.pdf]

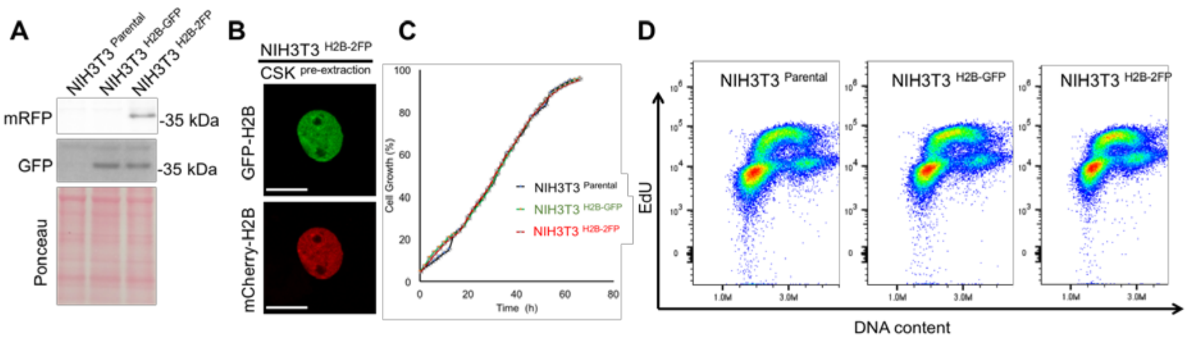

Fig. S1. Validations of NIH3T3 expressing fluorescently-tagged H2B. (A) Representative immunoblot analysis confirming the expression of H2B-GFP and H2B-mCherry using antibodies against GFP or mRFP (monomeric red fluorescent protein) in the derived NIH3T3 cell as indicated. (B) Validation of H2B-GFP and H2B-mCherry incorporation into chromatin using pre-extraction with CSK buffer prior to cell fixation (see methods), scale bar = 10 µm. (C) Quantitative analysis of the proliferation rates of the indicated NIH3T3 cell derivatives were using an automated live cell imaging system (Incucyte-ZOOM), data from one experiment performed in triplicates are presented as Mean±SE. (D) Flow-cytometry for cell cycle analysis (DNA content) and EdU incorporation (replication rate) showing similar profiles across NIH3T3 cell derivatives. Cells were pulsed labelled with EdU for 30 minutes, then fixed and permeabilised before staining for EdU (using Click-chemistry) and DNA (using propidium iodide).

Biology Open • Supplementary information

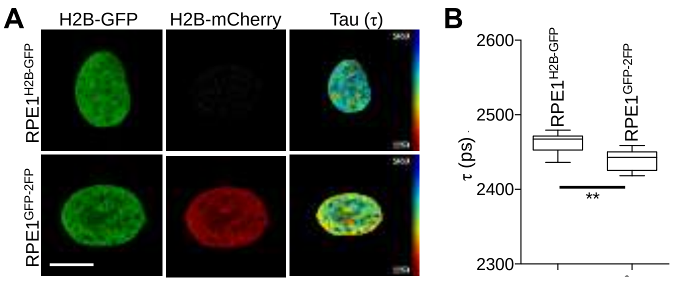

Fig. S2. Establishment of chromatin FLIM assay in RPE1 cells. (A) Examples of FLIM measurements in human RPE1 cells as indicated (H2B-GFP and H2B-mCherry images' scale bar = 10 μm). (B) Quantification of τ in multiple RPE1 cells as in (A), data expressed in picoseconds (ps) as Mean τ±SD, n≧10, ** indicates *p*=0.0012, Student's *t*-test.

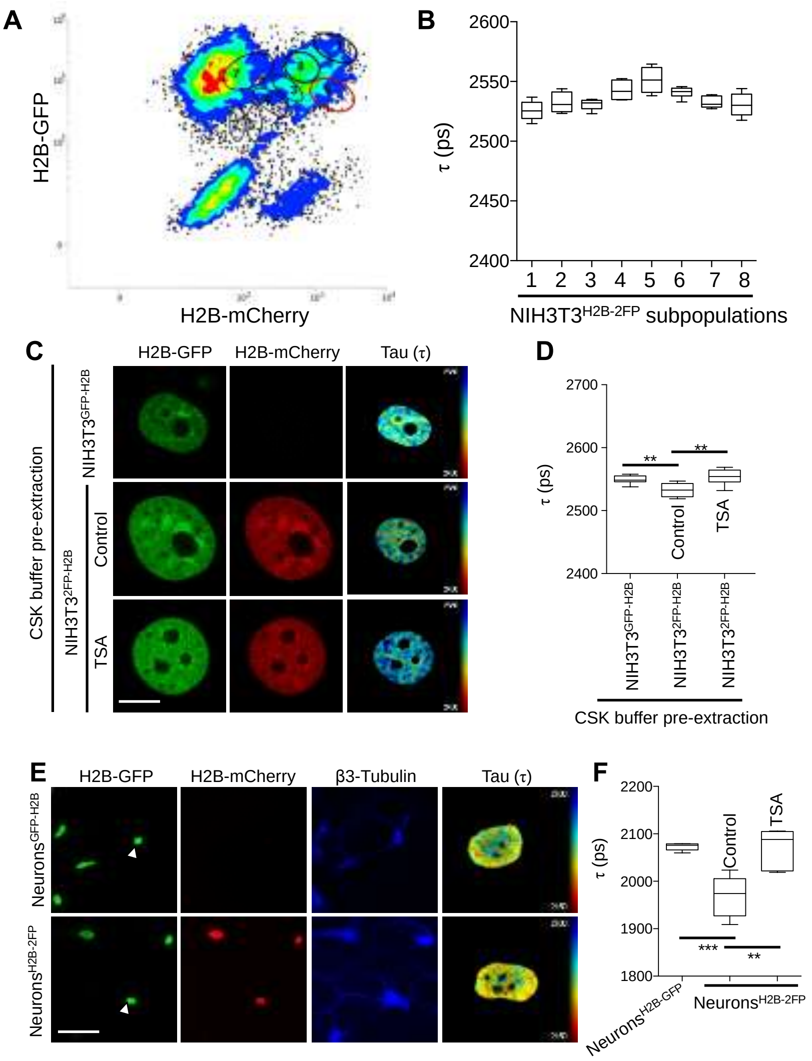

Fig. S3. Optimisations of chromatin FLIM for multi-cell analyses. (A) Two-dimensional scatter plot for NIH3T3 cells co-expressing H2B-GFP and H2B-mCherry (NIH3T3$^{H2B-2FP}$). The indicated numbers (1-8) correspond to FACS-sorted subpopulations of cells that express varying levels of H2B-GFP and mCherry-H2B. (B) Quantification of τ in multiple FACS-sorted subpopulations of NIH3T3$^{H2B-2FP}$ cells from (A) and data are expressed in picoseconds (ps) as Mean τ ± SD, n=5. (C) Representative examples of FLIM measurements conducted in the indicated cells and conditions after CSK-buffer pre-extraction followed by fixation (scale bar = 10 μm). (D) Quantification of τ data from (C), expressed in picoseconds (ps) as Mean τ±SD, n≧7, ** indicates p=0.0023 and 0.001, respectively, Student's t-test. (E) Representative examples of FLIM measurements conducted in hippocampal neurons after CSK-buffer pre-extraction and fixation (scale bar = 100 μm, the FLIM analysed nuclei are indicated by white arrows). (F). Quantification of τ data from C, expressed in picoseconds (ps) as Mean τ±SD, N=5, ** indicates p=0.0062, *** indicates p= 0.0007, Student's t-test.

Biology Open • Supplementary information

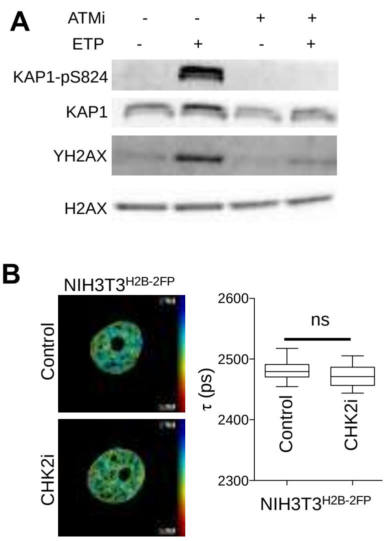

Fig. S4. Analysis of the inhibition of ATM-dependent signalling after DNA damage and the effect of CHK2 inhibition on chromatin structure. (A) Immuno-blot analysis of NIH3T3 treated as indicated confirming the inhibition of ATM-dependent signalling after DNA damage. **(B)** *Left panel*: example of GFP-H2B fluorescence lifetime in fixed NIH3T3$^{2FP\text{-}H2B}$ treated with CHK2i; *right panel*: corresponding quantifications, $n \geqq 16$, *ns* indicates *not-significant* p=0.075, Student's *t*-test.

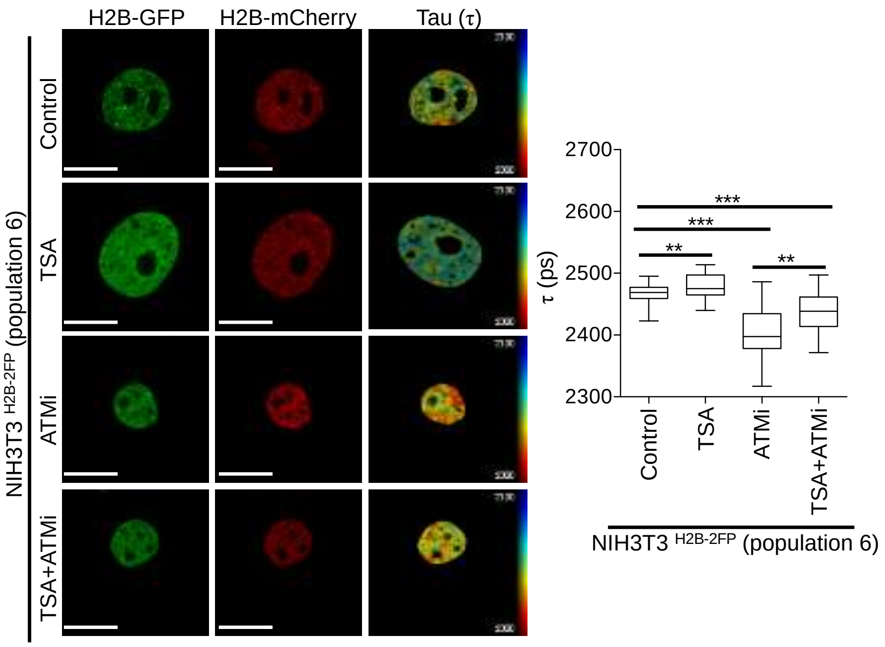

Fig. S5. Histone deacetylase inhibition does not completely alleviate higher chromatin compaction after ATM inhibition. Cells were treated with TSA (1 µM), or ATM (10 µM) inhibitor each alone or in combination for 16 hours, then followed by FLIM analysis after fixation. *Left panel* shows representative FLIM maps after these treatments with quantifications shown on the *rights panel*. Fluorescence lifetime (τ) data are presented as Mean τ ± SD, from three independent experiments, n≧ 25, ** indicates p=0.0082 and p=0.0011, respectively, *** indicates p＜0.001, Student's *t*-test.

Biology Open • Supplementary information
